# How to Solve the Time Dilemma? The Influence of Team Temporal Leadership on Team Innovation Performance

**DOI:** 10.3389/fpsyg.2021.634133

**Published:** 2021-07-22

**Authors:** Zhengqiao Liu, Xiliang Liu, Xianchun Zhang

**Affiliations:** ^1^Digital Economy Academy, Yango University, Fuzhou, China; ^2^School of Marxism, Hunan University, Changsha, China; ^3^Maritime Silk Road Tourism Economic Research Center, Guilin Tourism University, Guilin, China

**Keywords:** team temporal leadership, time pressure, team learning behavior, team innovation performance, conservation of resource theory

## Abstract

Time pressure (TP) is the most common kind of pressure faced by R&D teams. How to improve team innovation performance (TIP) when time resources are insufficient has been a concern of practitioners and scholars. The purpose of this paper is to put forward some suggestions to solve that time dilemma. We conducted a survey based on a sample of 163 teams. In the first-stage survey (time 1), we measured the team temporal leadership (TTL) and TP. In the second-stage survey (time 2), we measured team learning behavior (TLB). TIP was measured in the third-wave survey (time 3). The results are as follows: (i) TTL has a significant positive impact on the TLB and TIP; (ii) TLB plays a mediating role in the relationship between TTL and TIP; and (iii) TP can positively moderate the relationship between TTL and TLB, that is, the promoting effect of TTL on TLB is more pronounced under the higher level of TP. These findings reveal the influence mechanism of TTL on TIP from the perspective of TLB and TP.

## Introduction

“Time is money, efficiency is life”: the competition among modern enterprises has changed from the price competition to the time competition. Since the time factor plays a more important role in the survival and development of enterprises, they can seize the market opportunities only by developing the latest products in the shortest time. In recent years, scholars gravitate toward the role of the time factor in improving organizational competitiveness and conduct many beneficial explorations (George and Jones, 2000; Sonnentag, 2012). Modern enterprises that emphasize interaction and cooperation have paid more attention to team management mode. To specify, to manage the limited time resources, team leaders will incorporate time management into their leader behaviors, including time fragmentation management, time uncertainty management, and time ambiguity management, and thereby form team temporal leadership (TTL; Mohammed and Nadkarni, 2011; Maruping et al., 2015). However, team members often face a common and intense work pressure [i.e., time pressure (TP)]. When team members only cope with the TP by themselves, they may be to focus on their current tasks. If the leader can effectively help the team members resolve the dilemma of time shortage, the enterprise can gain more competitive advantages (Chen and Nadkarni, 2017; Chen and Liu, 2020; Zhang et al., 2021). Therefore, the influence of TTL on team innovation performance (TIP) and its internal mechanism is worthy of our attention.

Due to the fierce competition in the external environment, the organization needs to innovate continuously to maintain its competitive advantage. In such a dynamic context, learning is an important way for organizations to acquire knowledge and improve innovation performance (Koeslag-Kreunen et al., 2018; Watzek and Mulder, 2019; Deng et al., 2020). According to the literature, learning within a team can increase the knowledge, experience, and ability of members, and it may thus influence their innovation ability (Wang and Rode, 2010; García-Morales et al., 2012; Kim et al., 2020). Team learning behavior (TLB) can help team members to meet their development needs and help the team to achieve their goals by sharing knowledge and exchanging information among members (Gibson and Vermeulen, 2003; Widmann et al., 2016). Till now, the literature documents determinants of TLB encompassing team characteristics, like team climate and team cooperation (Guchait et al., 2017; Raes et al., 2017), as well as, leadership style, for instance, empowering leadership, shared leadership, and coordinated leadership (Durham et al., 1997; Wang et al., 2017; Koeslag-Kreunen et al., 2018). Due to the differences in working rhythm and time utilization of team members, time management is an inevitable issue in team management. By coordinating the personal time of members with the urgency of tasks, TTL can take measures to complete work tasks and increase individual leisure time in the meanwhile. In this way, team members can reach a consensus on time arrangement, which is beneficial to form a favorable climate for generating TLB (Mohammed and Nadkarni, 2011; Chen et al., 2018). However, whether TLB can play a mediating role in the relationship between TTL and TIP need further empirical evidence to support.

Since the rhythm of society is accelerating in the context of economic globalization, an increasing number of employees perceive insufficient time. Such subjective judgment is called TP, that is, the extent to which employees believe that they do not have sufficient time to complete multiple tasks. This kind of pressure is closely related to job requirements and work tasks (Bakker et al., 2014; Maruping et al., 2015). With the increasingly intensified competition of the external environment of the organization, organizational members are often required to complete a lot of work in a limited time. As a result, TP becomes the most common kind of work pressure experienced by individuals and teams, and it receives much attention from scholars and practitioners these years (Syrek et al., 2013). Undoubtedly, TTL can improve the way that teams and members allocate time resources. According to the resource allocation theory, the effective allocation of time resources can help team members better cope with TP (Merkel and Lohse, 2019; Sheng et al., 2019). Although scholars have studied the boundary conditions of the impact of TP on creativity from the perspectives of personality traits, job demands-resource model, and leadership. Temporal leadership is largely overlooked by academic research (Baer and Oldham, 2006; Sacramento et al., 2013; Maruping et al., 2015). Therefore, it is of great theoretical and practical significance to empirically explore whether TP can play a moderating role on the team level (i.e., TTL and TLB).

**FIGURE 1:**
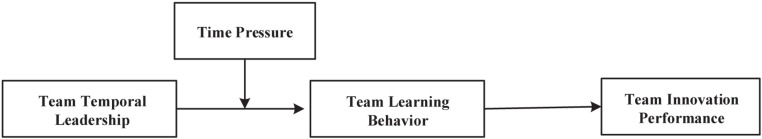
Research model.

In light of the above, this paper explores the relationship between TTL and TIP based on the conservation of resource theory (COR). Furthermore, this study tests the mediating effect of TLB and the moderating effect of TP on the above relationship. Figure 1 shows the theoretical model. We hope this paper can clarify the internal mechanism of the influence of TTL on TIP theoretically, and can help managers improve the level of TIP through better time management in practice.

## Theory and Hypotheses

### Team Temporal Leadership and Team Innovation Performance

The concept of temporal leadership originates from time, interaction, and performance theory. Initially, the literature defines it as an organizational mechanism that can alleviate time scarcity and utilize time diversification (Santos et al., 2016). However, this definition is relatively abstract since it is mainly used to explain organizational behaviors at the macro level. As academia emphasizes studies at the team level in recent years, the research on temporal leadership has been extended to the team level. For instance, McGrath and Rotchford (1983) argue that temporal leadership focuses on the core activities within the team. These activities allow teams to adjust external time parameters effectively, and thus improve their timeliness as well as performance. Although this argument does not specify who is responsible for executing time-related activities, many scholars believe the team leader is the person in charge. Therefore, they introduce the term “temporal leadership” to explain the time-related challenges faced by leaders (Ancona et al., 2001; Halbesleben et al., 2003; Chen et al., 2018). Furthermore, some researchers propose that it is inevitable for leaders to adjust different working rhythms and synchronize the working cycles of all members (Mohammed and Alipour, 2014; Maruping et al., 2015). From this point of view, temporal leadership includes three core elements, namely, scheduling, temporal synchronization, and temporal resources allocation. In detail, scheduling refers to specifying a clear timetable for the deadline of team activities; time synchronization includes making a chronological order and coordinating activities of different team members; time resource allocation means allocating time for team activities efficiently (especially when TP is high, Mohammed and Nadkarni, 2011; Yuan and Lo, 2018).

According to the COR, individuals with more resources are less vulnerable to resource loss and are more capable of resource access (Hobfoll, 1989; Halbesleben et al., 2014; Liu et al., 2020; Yao et al., 2020). The implementation of positive behaviors at work (such as innovative behaviors) depends on whether an individual has enough resources and can devote these resources to the work (Fritz et al., 2010; Montani et al., 2016). Through taking charge of time planning and arranging time reasonably, TTL can help team members save enough resources to perform innovative behaviors. Meanwhile, TTL can motivate team members to mobilize the resources they saved to innovative behaviors through supervision (e.g., urging team members to finish the task within the deadline). To specify, first, TTL can improve the concentration of the members. By scheduling time, leaders can develop a “task schedule” for members that can urge them to complete certain tasks before certain time nodes. Such a task schedule can help members save their time resources and benefit them by keeping these resources in store for performing innovation at work (Mohammed and Nadkarni, 2011; Maruping et al., 2015; Santos et al., 2016). Moreover, TTL helps to form a favorable working climate. By coordinating the time of the members actively, leaders can reduce conflicts among members caused by time problems. To ensure each member follows the time plan, TTL will encourage members with a light workload to help other colleagues after completing their tasks. This will help to create a supportive and friendly working climate and thereby generate innovative ideas (Gevers and Demerouti, 2013; Mohammed and Alipour, 2014; Maruping et al., 2015). Furthermore, TTL helps members to use time more effectively. By allocating time effectively, leaders can reduce unreasonable arrangements. Under their influence, team members can learn how to make time optimization, which can make members be more comfortable with the tasks and have more time to obtain resources besides the work. Through the effective transformation of resources they get, team members can perform innovative behaviors more easily. Based on the above analysis, this paper proposes the following hypothesis:

*Hypothesis 1: TTL is positively correlated with TIP.*

### The Mediating Effect of Team Learning Behavior

Team learning behavior (TLB) refers to the collective reaction and course of action in which members share, integrate, and apply task-related knowledge (Edmondson, 1999; Gibson and Vermeulen, 2003; Widmann et al., 2016). Through collective learning, knowledge and skills at the individual level can be interactive, amplified, and pooled, thereby producing common knowledge and skills at the team level. This shared knowledge enables all team members to better participate in solving problems, processing information, generating ideas, and evaluating results, and thus improving the TIP effectively (Leicher and Mulder, 2016; Widmann et al., 2016; Sun et al., 2017).

TTL can provide more resources to team members and thereby promote TLB. To specify, TTL can stimulate the learning needs of the members. Since team leaders can save more time resources and reduce performance losses caused by time factors via planning time effectively, the team will be motivated to pursue higher goals, and thus create a need for learning (Halbesleben et al., 2003; Mohammed and Nadkarni, 2011; Gevers and Demerouti, 2013). Moreover, TTL can create a good environment for TLB. According to the literature, the leadership style can influence the team climate, which in turn affects the learning willingness and learning ability of the members, and finally stimulate TLB (Gladstein, 1984; Gibson and Vermeulen, 2003). In particular, a coordinated leadership style can promote TLB. Since TTL is a kind of coordinated leadership, team members can work in an orderly manner following the coordination (Durham et al., 1997; Gevers and Demerouti, 2013). In addition, TTL can improve the learning efficiency of team members and facilitate knowledge sharing within the team through arranging time reasonably and coordinating the time of team members in the work process. This can promote the generation of TLB (Heo and Toomey, 2015; Elkjaer and Brandi, 2018), and finally help to improve TIP (Leicher and Mulder, 2016; Widmann et al., 2016; Sun et al., 2017). Based on the above analysis, this paper proposes the following hypothesis:

*Hypothesis 2: TLB mediates the relationship between TTL and TIP.*

### The Moderating Effect of Time Pressure

TP refers to the subjective perception of the individual that he or she does not have enough time to complete work tasks (Bakker et al., 2014; Maruping et al., 2015; Kocher et al., 2019). While long-term TP will affect the health and job performance of members negatively, some scholars believe that short-term TP may have a positive impact on job performance since it will stimulate the work potential of the members (Baethge et al., 2019; Maqbool et al., 2019; Peng et al., 2019). In fact, TP can be divided into challenge TP and hindrance TP (Chong et al., 2011). Challenge TP implies that when employees are completing challenging tasks arranged by their superiors, some challenging factors (e.g., complexity and importance of work) make them feel the pressure of time resource constraints; hindrance TP implies that employees feel the pressure of time shortage due to some hindrance factors (e.g., work conflict) when they are completing assigned tasks (Chong et al., 2011). Given the positive effects of challenge TP, only this type of TP is considered in this paper. Therefore, TP has two sides. On the one side, appropriate TP can teach team members to deal with urgent and hard tasks, which can benefit them to enhance their work competence in completing work challenges (Kühnel et al., 2012; Reis et al., 2016; Baethge et al., 2019). On the other side, the ability of time management is not natural. When team members are under high TP, they are more likely to seek the help of leaders who have rich experience and the power to decide the task progress. Therefore, it is particularly important to discuss how to help team members relieve TP from the perspective of leaders.

According to COR, when TP is high, leaders will allocate time resources actively. If the work arrangements of the members can be more clear, they can save their time resources and use these resources in team learning and knowledge sharing (Maruping et al., 2015; Baethge et al., 2019). Moreover, due to the shortage of time resources, leaders will prioritize work tasks and set clear objectives to prevent time from being wasted (Mohammed and Nadkarni, 2011; Maruping et al., 2015; Santos et al., 2016). In this situation, the task progress and time arrangement are under the control of the leader, which can align all the team members at the same time rhythm. This synchronous rhythm can alleviate the psychological pressure of the members greatly, and thus create good conditions for TLB. Furthermore, when TP is high, leaders will keep in close contact with team members to make full use of time resources. This helps to create a good working climate (Pearsall et al., 2009; Nielsen et al., 2012) and thereby promotes TLB (Gladstein, 1984; Gibson and Vermeulen, 2003). In contrast, when TP is low, the time resources are sufficient, making the average time of each member’s subtask longer and the degree of task refinement lower. The team members may have limited concentration during the work and can hardly learn skills in completing the task, which is not conducive to the generation of TLB. Moreover, since the clarity of priorities is low, team members may have the psychological burden of not being able to identify priorities, and this has an inhibiting effect on their knowledge sharing and learning behaviors. Based on the above analysis, this paper proposes the following hypothesis:

*Hypothesis 3: TP will positively moderate the relationship between TTL and TLB, that is, under the higher level of TP, TTL has a stronger promoting effect on TLB.*

## Materials and Methods

### Sample and Procedures

High-tech enterprises attach great importance to the learning and innovation of team members. Meanwhile, they are also under great TP. Since the Chinese government has paid attention to the development of the new energy industry in recent years, this industry has attracted a large number of high-tech enterprises, which benefits our investigation. The R&D team is the basic unit of high-tech enterprises to carry out innovative activities. Since it undertakes the largest amount of innovation tasks in the organization, R&D staff normally have limited time resources. Therefore, the R&D team is a representative research object of this study. The samples were from Shanghai, Guangzhou, and Shenzhen in China. Before distributing the questionnaire survey, we communicated with senior managers, human resource managers, and team leaders to obtain information such as the number of members in the investigated team, and then we prepared the corresponding number of questionnaires for each team. The questionnaire was filled out anonymously by the team members. In the first-stage survey (time 1), the team leader distributed the first part of the questionnaire to collect data including TTL, TP, and demographic variables. A month later (time 2), the team leader distributed the second part of the questionnaire to the team members and finished the measurement of TLB. After another 1 month (time 3), we surveyed team leaders and asked them to fill in the questionnaire of TIP. The questionnaire was sealed immediately after being filled out, and the respondents were numbered to ensure that the questionnaire could be matched successfully. In sum, we surveyed 250 teams and distributed 500 sets (1000 copies) of questionnaires. After eliminating unqualified questionnaires, 163 effective teams and 666 effective questionnaires were obtained, with an effective response rate of 66.60%. Among the evaluated team members, 51.50% were male and 48.50% female. In terms of age, 24.47% were aged 25 or below, 33.03% were aged between 26 and 35, 29.43% were aged between 36 and 45, and 13.07% were aged 45 or above. Regarding the educational background, 24.47% were junior college or below, 52.10% were undergraduate, 23.43% were graduate or above. Concerning the number of team members, teams with three or fewer members account for 10.96%, four to five members account for 30.03%, five to seven members account for 36.19%, and eight members or above account for 22.82%. With regards to years of working experience, 47.90% for less than 2 years, 28.23% for 3–5 years, 10.36% for 6–8 years, and 13.51% for more than 8 years.

### Measures

The scales used in this paper are obtained from international authoritative journals and have been recognized by many scholars. All scales involving variables are Likert five-point scale ranging from 1 “strongly disagree” to 5 “strongly agree.” The scale sources are as follows.

#### Team Temporal Leadership

This study adopted the seven-item scale developed on the basis of Ancona et al. (2001) and Mohammed and Nadkarni (2011). A sample item is “leaders often remind members of the deadline of important projects.”

#### Time Pressure

Five items used by Van Emmerik and Jawahar (2006) were used to measure TP. A sample item is “I think team members are pressed for time.”

#### Team Learning Behavior

Four items used by van Der Vegt and Bunderson (2005) were adopted in this study. A sample item is “our team members can positively comment on each other’s work to improve performance.”

#### Team Innovation Performance

A four-item scale used by Islam et al. (2009) was used to measure TIP. A sample item is “the team’s R&D activities can be completed within the expected time.”

#### Control Variables

Based on the research results of Mohammed and Nadkarni (2011), the gender, age, educational background, team scale, and working age in team were controlled.

### Aggregate Analysis

Since the TTL, TP, and TLB measurements are filled out by team members and aggregated to the team level, we need to examine intragroup homogeneity and intergroup differences to confirm the validity of data. Therefore, this study uses ICC(1), ICC(2), and R_wg_ to test the appropriateness of aggregating TTL, TP, and TLB of team members into the team level. The results of one-way ANOVA show that there are significant differences in both intergroup variance and intragroup variance of TTL [*F* = 3.48, *p* < 0.01; ICC(1) = 0.15; ICC(2) = 0.73; R_wg_ = 0.84], TP [*F* = 2.36, *p* < 0.01; ICC(1) = 0.32; ICC(2) = 0.79; R_wg_ = 0.91], and TLB [*F* = 2.68, *p* < 0.01; ICC(1) = 0.26; ICC(2) = 0.75; R_wg_ = 0.82]. Hence, the intragroup correlation coefficients ICC(1) and ICC(2) of TTL, TP, and TLB are all within an acceptable range, indicating that the aggregation of data at the team level is appropriate and effective.

## Results

### Descriptive Statistics

Table 1 reports descriptive statistics of each variable. Cronbach’s α values of the four variables are all greater than 0.70, indicating the high reliability of the scales. Moreover, TTL has significant positive correlation with TLB (*r* = 0.567, *p* < 0.01) and TIP (*r* = 0.406, *p* < 0.01), respectively, and TLB has significant positive correlation with TIP (*r* = 0.591, *p* < 0.01). Although this cannot fully support our hypothesis, it can provide preliminary support.

**TABLE 1:** Mean, standard deviation and correlation coefficient matrix.

	Sex	Age	Education	Scale	Working time	TTL	TP	TLB	TIP
Sex	–								
Age	–0.091	–							
Education	0.053	0.150	–						
Scale	–0.094	0.108**	0.187*	–					
Working age	0.026	–0.129	–0.002	−0.164*	–				
TTL	–0.007	–0.039	–0.013	0.054	–0.154	0.860			
TP	–0.097	0.021	–0.026	0.077	−0.257**	0.550**	0.858		
TLB	–0.030	0.022	0.064	0.077	–0.149	0.567**	0.669**	0.892	
TIP	–0.096	–0.127	–0.065	–0.017	–0.075	0.406**	0.504**	0.591**	0.853
Mean	0.485	2.307	1.988	2.709	1.896	3.671	3.740	3.540	3.164
SD	0.501	0.983	0.694	1.290	1.058	0.554	0.667	0.775	0.639

n1 = 163, n2 = 503, **p < 0.01, *p < 0.05, two-tail test, the diagonal is the value of Cronbach’s alpha.

### Measurement Model

To verify the discriminant validity between TTL, TP, TLB, and TIP, this study conducts confirmatory factor analysis on key variables and compares all models (Table 2). The results show that the fitting degree of the four-factor model (χ^2^/df = 1.837, IFI = 0.925, TLI = 0.911, CFI = 0.924, RMSEA = 0.072) is significantly better than that of alternative plausible models, demonstrating that the four-factor model has good discriminating validity and can better represent the factor structure of the measurement model.

**TABLE 2:** Confirmatory factor analysis results.

Models	Factors	x^2^	df	X^2^/df	IFI	TLI	CFI	RMSEA
Four–factor model	TTL, TP, TLB, TIP	301.268	164	1.837	0.925	0.911	0.924	0.072
Three–factor model	TTL + TP, TLB, TIP	376.752	167	2.256	0.885	0.867	0.884	0.088
Two–factor model	TTL + TP, TLB + TIP	529.646	169	3.134	0.803	0.774	0.800	0.115
Single–factor model	TTL + TP + TLB + TIP	645.830	170	3.799	0.740	0.704	0.736	0.131

Since four variables including TTL, TP, TLB, and TIP are all from the self-evaluation of members and leaders, it is difficult to eliminate common variance. Hence, we conduct Harman single factor test and find that the first factor without rotation explained only 24.291% of the total variation, accounting for less than 40%. Moreover, Table 2 illustrates that the single-factor goodness of fit model is significantly worse than that of the four-factor model, which means, there is no serious common method variation problem in this study (Podsakoff et al., 2003).

### Hypothesis Testing

#### Main Effect Test

This paper uses SPSS 23.0 software to conduct a stepwise regression analysis, and the results are shown in Table 3. According to M2 and M5, TTL is significantly positively correlated with TLB (*b* = 0.782, *p* < 0.001) and TIP (*b* = 0.450, *p* < 0.001); thus hypothesis 1 is supported.

**TABLE 3:** Regression analysis results.

		TLB			TIP	
	M1	M2	M3	M4	M5	M6
**Control variables**						
Sex	–0.043	–0.040	0.031	–0.127	–0.125	–0.107
Age	–0.056	0.013	0.012	−0.153*	–0.113^+^	−0.119*
Education	0.066	0.076	0.084	–0.046	–0.040	–0.074
Scale	0.054	0.006	0.003	0.068	0.040	0.037
Working age	–0.105^+^	–0.043	0.022	–0.049	–0.013	0.006
**Independent variable**						
TTL		0.782***	0.399***		0.450***	0.199**
**Mediating variable**						
TLB						0.449***
**Moderating variable**						
TP			0.605***			
**Interactive items**						
TTL×TP			0.115*			
R^2^	0.031	0.332	0.513	0.046	0.193	0.391
ΔR^2^		0.301***	0.181***		0.147***	0.198***

*n1* = 163, n2 = 503, ***p < 0.001, **p < 0.01, *p < 0.05, ^+^p < 0.1.

#### Mediating Effect Test

According to M6, there is a significant positive correlation between TLB and TIP (*b* = 0.449, *p* < 0.001). To further verify the mediating effect of TLB, this paper conducts bootstrapping mediating effect test through the SPSS23.0 software PROCESS plug-in. As shown in Table 4, the confidence interval (Bia-corrected 95%) for the indirect effect of TTL on TIP through TLB is [0.229, 0.491], excluding 0. This result demonstrates that the mediating effect of TLB between TTL and TIP exists, which verifies hypothesis 2.

**TABLE 4:** The mediating effect test.

Variables	Bia-Corrected 95%CI		Percentile 95%CI	
		
	Lower	Upper	Lower	Upper
**Bootstrapping**				
	Indirect effect			
TTL→TLB→TIP	0.229	0.491	0.227	0.488
	Direct effect			
TTL→TLB→TIP	–0.055	0.296	–0.055	0.296

**n1 = 163, n2 = 503, Bootstrapping for 5,000 random sampling.**

#### Moderating Effect Test

**FIGURE 2:**
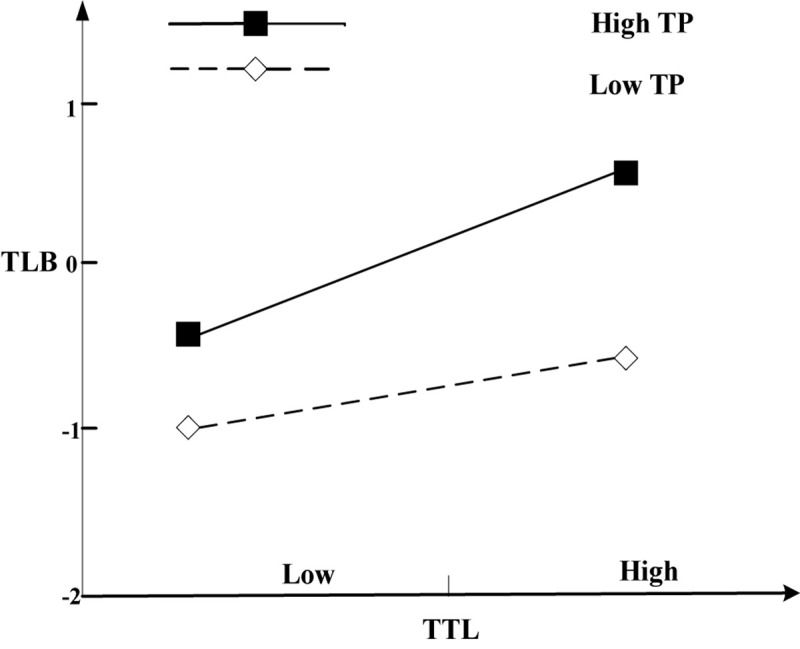
The moderating effect of time pressure.

Table 3 shows that the interaction terms of TTL and TP are significantly positively correlated with TLB (M3, *b* = 0.115, *p* < 0.05). Moreover, the simple slope test illustrates that TTL has a significant effect on TLB under high TP (mean +1 SD) (*b* = 0.416, *t* = 1.714, *p* < 0.1) whereas it has no significant effect under low TP (mean − 1 SD) (*b* = 0.262, *t* = 1.463, *p* = 0.145). Therefore, TTL is more likely to affect TLB under high TP than under low TP, and hypothesis 3 is supported. The simple slope test is shown in Figure 2.

## Discussion

Drawing on COR, this paper aims to examine the influence mechanism of TTL on TIP. Consistent with our conjecture, the mediating effect of TLB and the moderating effect of TP exist. By analyzing the data from 163 R&D teams of high-tech enterprises, we find that TTL has a significant positive impact on the TLB and TIP. We further find that TLB plays a mediating role in the relationship between TTL and TIP. Finally, TP can positively moderate the relationship between TTL and TLB. More specifically, under the higher level of TP, TTL will have a stronger promoting effect on TLB.

### Theoretical Implications

Our study makes several contributions to the literature. First, this paper extends research frontiers of the theory about leadership by focusing on time management at the team level in the Chinese context. Among previous studies on the influence of team leadership on team behavior, scholars rarely integrate the time factor into the constructs of team leaders (Shamir, 2011; Santos et al., 2016). Moreover, the existing theory is not convincing enough to explain the relationship between the treatment of TP leaders and team behavior (Bluedorn and Jaussi, 2008; Maruping et al., 2015). Our paper incorporates the time factor into the constructs of team leaders, underscores the guiding role of temporal leadership in team behavior, and consequently enables teams to work effectively under the TP. The results show that TTL can affect TLB and TIP positively, which reflects the important role of TTL in effective team management. In addition, while China is one of the countries with most populous as well as the fastest economic development where team members often face huge time pressure, temporal leadership in Chinese context is extremely scarce (Chen and Liu, 2020; Zhang et al., 2021). Therefore, our findings expand the application of temporal leadership in Chinese contexts and enrich the related studies on temporal leadership in different contexts.

Second, this paper explores a new path between TTL and TIP. Previous studies mainly take TTL as a direct predictor of the team process, team time cognition, and other variables, but pay less attention to the mediating mechanism between TTL and TIP (Mohammed and Nadkarni, 2011; Mohammed and Alipour, 2014; Maruping et al., 2015). Drawing on COR, this paper argues that team leaders can help team members obtain more time resources by planning time of the members and determining the priority of tasks. By this means, team members can devote these time resources to team learning, thereby improving the innovation performance. Therefore, our finding expands the application scope of COR and offers new insight into the mechanism of TTL.

Third, we find the moderating effect of TP on the relationship between TTL and TLB. The existing literature has often ignored the boundary conditions of the effectiveness of TTL as well as the effect of TP (Maruping et al., 2015; Maqbool et al., 2019). However, TP is one of the most common objective pressure faced by team members which runs through the whole process of completing tasks (Mohammed and Nadkarni, 2011; Mohammed and Alipour, 2014). Hence, it is necessary to consider the moderating effect of TP. Based on the COR, this paper takes TP as the boundary condition of the relationship between TTL and TLB. We find that in the case of high TP, TTL has a stronger positive effect on TLB. This finding could provide scholars and practitioners with a new way of improving TLB.

### Practical Implications

This paper also bears implications in practice. First, leaders should make time management active and effective. Managers tend to focus on people, money, and materials in daily work, while paying less attention to time. Such imbalance will have an inhibiting effect on completing tasks on time and of high-quality. Therefore, the leader should actively coordinate an allocate the time resource of team members, remind members of deadlines regularly, and help them make time plans. Moreover, leaders should help team members to solve their time problems and improve working efficiency. Making use of saved time to learn new knowledge and share knowledge actively, TIP can be improved. Finally, leaders can exert team TP appropriately. Since moderate TP can strengthen the effective time planning of the team leaders, they can save more time resources. Similarly, using these time resources to learn and share new knowledge can promote TIP.

### Limitations and Future Research Direction

Our study also has some limitations. First, although we collect data in three-time points and match the data between team leaders and team members to reduce common method variation, this problem cannot be eliminated. In future studies, the combination of experimental method and experience sampling method can be considered to further improve the accuracy of research results. Second, while many scholars call for studies on the mediating mechanism between temporal leadership and innovation performance in the Chinese context, this paper only discusses the mediating role of TLB. In the follow-up research, more key transmission mechanisms such as team reflection, shared time cognition and team knowledge search. Third, since the pace of society is accelerating, organizations are facing greater TP than individuals and teams. Therefore, future studies may discuss TP at the organizational level. Fourth, we have only focused on challenge time pressure; however, what the role of hindrance time pressure might play in our theoretical model remains unexplored. Therefore, in future studies, we can incorporate hindrance time pressure into our theoretical model. Finally, at the beginning of the research design, we try to use proactive personality as the boundary condition in the relationship between TTL and TLB. However, it has not received support. In the future, another personal trait can be discovered to enrich the boundary condition of the above relationship.

## Data Availability Statement

The original contributions presented in the study are included in the article/supplementary material, further inquiries can be directed to the corresponding author/s.

## Ethics Statement

The studies involving human participants were reviewed and approved by Academic Ethics Committee of Yango University. The patients/participants provided their written informed consent to participate in this study. Written informed consent was obtained from the individual(s) for the publication of any potentially identifiable images or data included in this article.

## Author Contributions

XZ conceived, designed the study, and completed the manuscript in English. ZL participated in drafting the article, revised it critically for important intellectual content, gave many good research advices, and revised the manuscript. XL was responsible for data collection and other supporting work. All authors contributed to the article and approved the submitted version.

## Conflict of Interest

The authors declare that the research was conducted in the absence of any commercial or financial relationships that could be construed as a potential conflict of interest.
